# Improvement of Endovascular Stroke Treatment: A 24-Hour Neuroradiological On-Site Service Is Not Enough

**DOI:** 10.1155/2018/9548743

**Published:** 2018-01-04

**Authors:** Omid Nikoubashman, Kolja Schürmann, Ahmed E. Othman, Jan-Philipp Bach, Martin Wiesmann, Arno Reich

**Affiliations:** ^1^Department of Diagnostic and Interventional Neuroradiology, University Hospital, RWTH Aachen University, Pauwelsstr. 30, 52074 Aachen, Germany; ^2^Department of Neurology, University Hospital, RWTH Aachen University, Pauwelsstr. 30, 52074 Aachen, Germany; ^3^Department of Radiology, University Hospital Tübingen, Hoppe-Seyler-Straße 3, 72076 Tübingen, Germany

## Abstract

**Background and Purpose:**

With the advent of endovascular stroke treatment (EST) with mechanical thrombectomy, stroke treatment has also become more challenging. Purpose of this study was to investigate whether a fulltime neuroradiological on-site service and workflow optimization with a structured documentation of the interdisciplinary stroke workflow resulted in improved procedural times.

**Material and Methods:**

Procedural times of 322 consecutive patients, who received EST (1) before (*n* = 96) and (2) after (*n* = 126) establishing a 24-hour neuroradiological on-site service as well as (3) after implementation of a structured interdisciplinary workflow documentation (“Stroke Check”) (*n* = 100), were analysed.

**Results:**

A fulltime neuroradiological on-site service resulted in a nonsignificant improvement of procedural times during out-of-hours admissions (*p* ≥ 0.204). Working hours and out-of-hours procedural times improved significantly, if additional workflow optimization was realized (*p* ≤ 0.026).

**Conclusions:**

A 24-hour interventional on-site service is a major prerequisite to adequately provide modern reperfusion therapies in patients with acute ischemic stroke. However, simple measures like standardized and focused documentation that affect the entire interdisciplinary pre- and intrahospital stroke rescue chain seem to be important.

## 1. Introduction

As endovascular stroke treatment (EST) with mechanical thrombectomy has been established as a common treatment option for large vessel occlusion (LVO) acute ischemic stroke (AIS), stroke treatment has also become more challenging [1–6]. EST is based on a tightly coordinated interdisciplinary sequence of prehospital and in-house procedures that require an optimal workflow and manpower, especially at night or during weekends [7–9]. Almekhlafi et al. reported that in-house procedures for EST take significantly longer time if patients are admitted out-of-hours [10, 11]. After a retrospective analysis of our procedural times in early 2012, we undertook various precautionary measures in order to improve procedure times and to avoid a possible out-of-hours effect. The most substantial countermeasures were (1) a fulltime neuroradiological on-site service and (2) consecutive workflow optimization with stringent interdisciplinary documentation, especially of key time intervals. We prospectively analysed the effects of these two countermeasures in order to investigate whether a fulltime neuroradiological on-site service and implementation of a structured interdisciplinary workflow documentation resulted in improved procedural times.

## 2. Material and Methods

### 2.1. Patients

Our analysis is based on a prospective observational registry of all consecutive patients of the University Hospital RWTH Aachen (Aachen, Germany) tertiary stroke centre, who received any kind of reperfusion therapy for AIS between February 2010 and March 2015. We chose this timeframe to allow for comparable sample sizes in our subgroups (see below). Between February 2010 and March 2015, 1009 patients with AIS received reperfusion therapy. We excluded 70 cases of in-house strokes from our study. This left 939 patients with a community-onset stroke. Of those 337 were transferred to the angiography suite for EST. After interdisciplinary neurological-neuroradiological treatment decision, 15 of the 337 patients did not receive EST and were excluded from our subgroup analysis. This left 322 patients, who received EST in our institution and who were included in our study.

**Figure 1:**
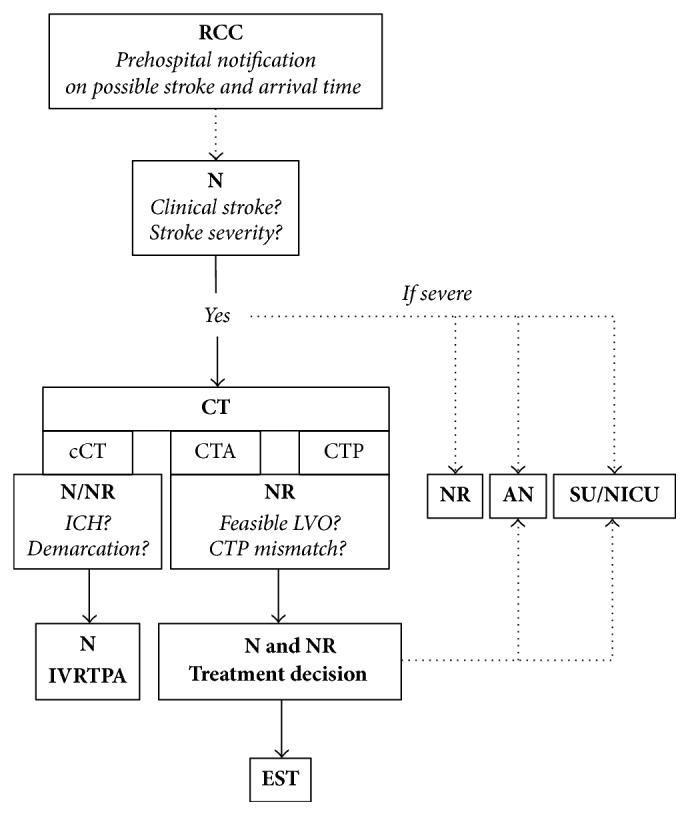
Schematic illustration of preinterventional workflow in our institution. RCC: rescue coordination centre; N: neurologist; NR: neuroradiologist; AN: anaesthesiologist; SU/NICU: stroke unit/neurological intensive care unit; CT: computed tomography; cCT: cranial CT; CTA: CT angiography; CTP: CT perfusion; ICH: intracranial/intracerebral haemorrhage; LVO: large vessel occlusion; IVRTPA: systemic thrombolysis; EST: endovascular stroke treatment. Dotted lines: phone calls. After the neurologist in charge is informed about a possible stroke by the rescue coordination centre, the neurologist informs the neuroradiologist in charge about the case. If a short clinical examination confirms the stroke, the anaesthesiologist on call is also informed and the patient is transferred to the CT suite, where the extent of stroke is assessed and an unenhanced CT is performed. In the meantime, the stroke unit or neurological intensive care unit is informed about the case. If there is no haemorrhage, and the patient fulfils standard inclusion criteria for thrombolysis, systemic thrombolysis is administered and a CT angiography is performed. If there is occlusion of a large cerebral artery, the interventionalist and the anaesthesiologist are informed and the patient is transferred to the angiography suite. In the angiography suite, there is parallel workflow with the interventionalist performing the groin puncture, while the anaesthesiologist intubates the patient.

**Figure 2:**
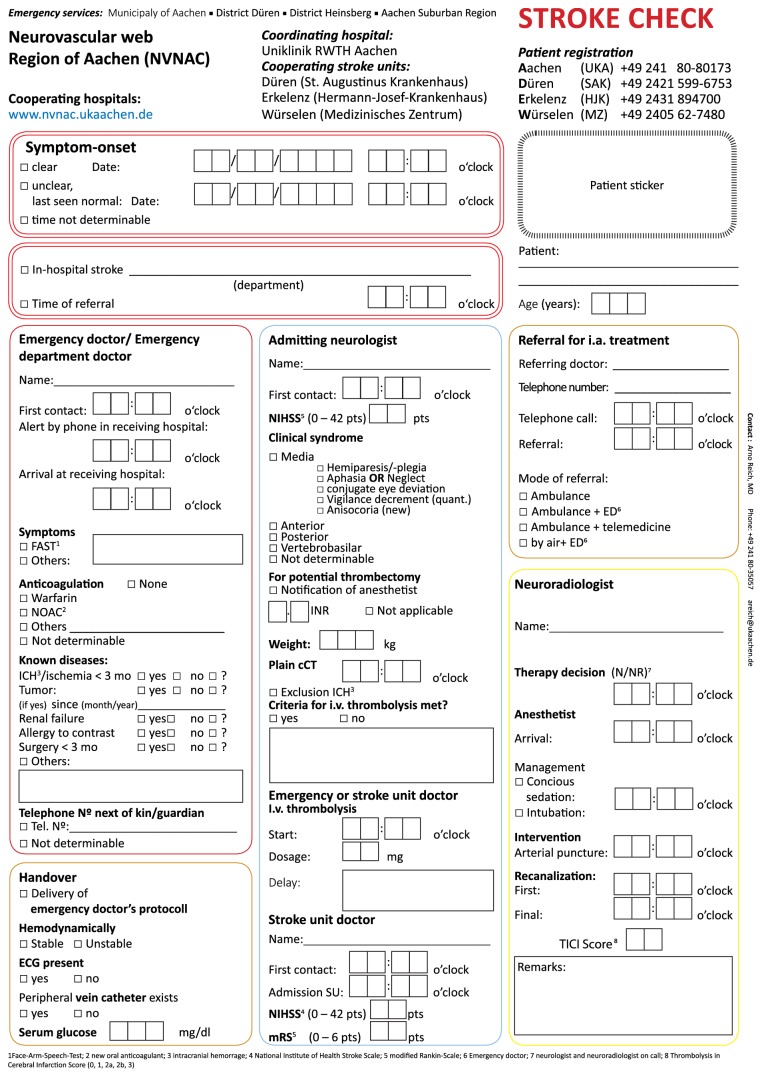
“Stroke Check” form for interdisciplinary documentation of treatment related data. Initial NIHSS scores and follow-up mRS are documented on the back of the sheet.

In order to determine the effect of a fulltime neuroradiological on-site service and workflow optimization with extensive documentation of procedural times, we compared procedural times of three phases: (1) phase 0: all patients, who were admitted before establishing a 24 h neuroradiological on-site service in May 2012, (2) phase 1: all consecutive patients, who were admitted after establishing a 24 h neuroradiological on-site service but before workflow optimization with extensive documentation of procedural times in February 2014, (3) phase 2: all remaining consecutive patients after workflow optimization (Figure 1) with extensive documentation of procedural times (“Stroke Check,” Figure 2).

### 2.2. Procedures and Workflow

The cornerstones of acute stroke treatment in our institution are (1) the neurological stroke team with vascular-experienced neurologists on-site 24 hours a day in the emergency department, (2) the supraregional comprehensive stroke unit (SU), (3) the neurological intensive care unit (NICU), and (4) the interventional neuroradiological team that is on-site during working-hours and on call (in a radius of 30 minutes) the rest of the time.

The rescue coordination centre informs the neurologist in charge about a stroke and the neurologist informs the neuroradiologist before the patient's arrival in the emergency room. Only if a short clinical examination confirms the stroke, the anaesthesiologist in charge is also informed and the patient is transferred to the CT suite, where an unenhanced CT is performed. Next, in the absence of medical contraindications, systemic thrombolysis is administered, followed by CT angiography and a CT perfusion. Alternatively, magnetic resonance imaging (MRI) is performed as first- or second-line imaging if CT imaging is (expected to be) nonconclusive. A patient is regarded as eligible for EST when there is clinical stroke, absence of haemorrhage or large infarction, and proven and accessible LVO. In addition, salvageable brain tissue (mismatch between cerebral blood volume and cerebral blood flow on CT perfusion imaging) is also considered whenever other criteria for EST are ambiguous (for example, in wake-up strokes). EST can be initiated after the time window of 4.5 hours if cranial imaging indicates that there is clinically relevant salvageable brain tissue. In every case, the interventional neuroradiologist and an experienced vascular neurologist (on call) discuss and decide about the therapy. Decision-making is based on medical and social criteria. Whenever possible the patient and/or the patient's relatives are involved in the decision-making process. If the decision to perform EST is made, the patient is transferred to the angiography suite. As all endovascular procedures are performed using general anaesthesia, there is parallel workflow with the interventionalist performing the groin puncture, while the anaesthesiologist intubates the patient. Standard endovascular treatment with and without stent retrievers is performed as reported previously [12].

### 2.3. Clinical, Procedural, and Radiological Data

After obtaining permission from our local ethics board, we assessed demographical data, clinical presentation (NIHSS) and disability (mRS) on admission, and disability at follow-up (mRS at discharge and day 90), cerebrovascular risk factors and primary as well as prophylactic use of antiplatelet/anticoagulant medication, and ischemic stroke aetiology (adapted from TOAST) [13]. Two neuroradiologists, who were blinded to clinical data, evaluated radiological data. A reference standard was established for statistical analyses in a consensus reading. Radiological and procedural data comprised initial and postinterventional/follow-up imaging with site of LVO and extent of initial ischemic changes (ASPECTS), type of treatment (including systemic therapy), procedural time intervals, and result of recanalization (TICI) [14, 15]. Door-to-image time was defined as the time between the documented time of admission in the emergency department and completion of the first cerebral imaging with computed tomography or magnetic resonance imaging. Image-to-puncture time was defined as the time between completion of the first cerebral imaging with CT or MRI to groin puncture in the angiography suite. Puncture-to-revascularization time was defined as the time between groin puncture in the angiography suite and first revascularization of the affected vessel (relative TICI improvement ≥ 1). Primary outcome measures were procedural times as defined above and functional outcome defined as mRS of ≤2 after 90 days.

### 2.4. Statistical Analysis

Pearson's *χ*^2^ tests and Fisher's exact tests were used whenever applicable. Student's *t* tests, Mann–Whitney *U* tests, and analysis of variance (ANOVA) were used for comparison of continuous data after testing for normal distribution with a Shapiro-Wilk test. *p* values under the alpha level of 0.05 were defined as significant. All statistical analyses were performed with SPSS 23 software (IBM, Armonk, New York).

## 3. Results

**Figure 3:**
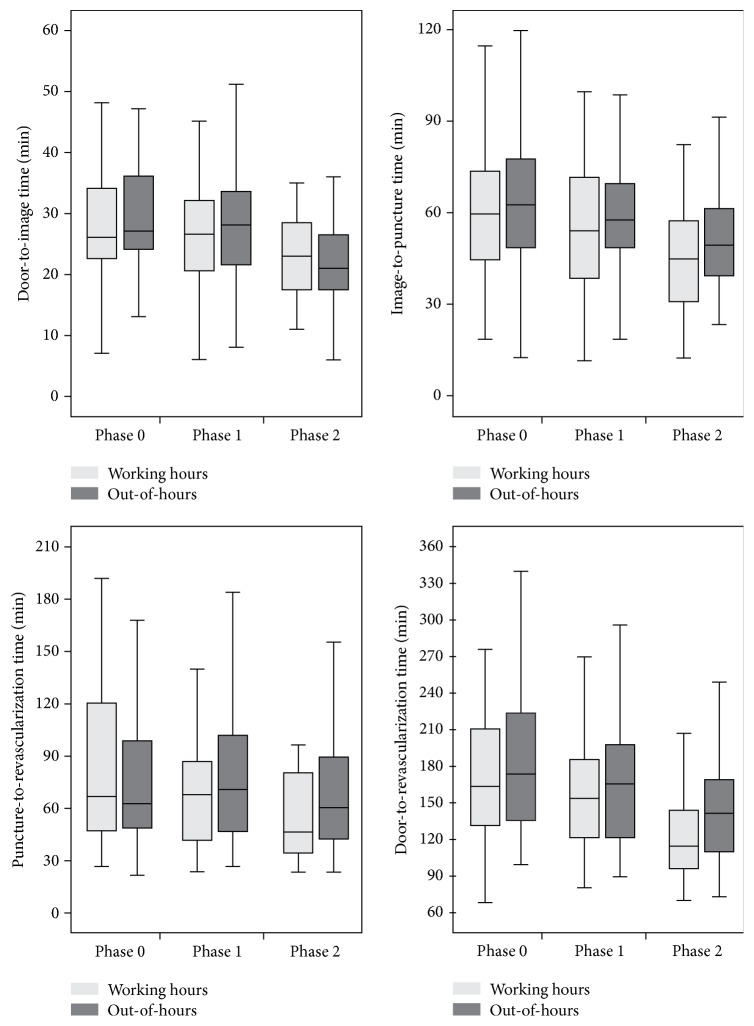
Boxplots illustrating procedural times. Outliers are not illustrated. Phase 0: before establishing a 24 h neuroradiological on-site service in May 2012. Phase 1: after introduction of a 24 h neuroradiological on-site service but before workflow optimization with extensive documentation of procedural times in February 2014. Phase 3: after workflow optimization with extensive documentation of procedural times.

Ninety-six patients received interventional treatment before establishing a 24-7 neuroradiological service (phase 0). Fifty-three (55%) of these patients were admitted out-of-hours. Procedural times of patients admitted in working-hours and out-of-hours did not differ significantly. Median intervals for working-hours versus out-of-hours admissions were as follows: door-to-image: 26 versus 26 minutes (*p* = 0.405), image-to-puncture: 58 versus 61 minutes (*p* = 0.448), puncture-to-revascularization: 65 versus 61 minutes (*p* = 0.811), and door-to-revascularization: 161 versus 171 minutes (*p* = 0.380) (Figure 3). Working-hours versus out-of-hours admissions did not differ significantly with regard to favourable functional outcome rate (15/37 versus 16/42; *p* = 0.824) at 90 days.

One hundred twenty-six patients received interventional treatment after introduction of a fulltime neuroradiological on-site service but before workflow optimization with extensive documentation of procedural times (phase 1). Seventy (56%) of these patients were admitted out-of-hours. Procedural times of patients admitted in working-hours and out-of-hours did not differ significantly. Median intervals for working-hours versus out-of-hours admissions were as follows: door-to-image: 26.5 versus 28 minutes (*p* = 0.418), image-to-puncture: 52.5 versus 56 minutes (*p* = 0.124), puncture-to-revascularization: 66 versus 69 minutes (*p* = 0.506), and door-to-revascularization: 151 versus 163 minutes (*p* = 0.214) (Figure 3). Working-hours versus out-of-hours admissions did not differ significantly with regard to favourable functional outcome rate (14/51 versus 20/63; *p* = 0.618) at 90 days.

One hundred patients received interventional treatment after workflow optimization with extensive documentation of procedural times (phase 2). Sixty-three (63%) of these patients were admitted out-of-hours. Procedural times of patients admitted in working-hours and out-of-hours did not differ significantly. Median intervals for working-hours versus out-of-hours admissions were as follows: door-to-image: 23 versus 21 minutes (*p* = 0.526), image-to-puncture: 43.5 versus 48 minutes (*p* = 0.109), puncture-to-revascularization: 45 versus 59 minutes (*p* = 0.214), and door-to-revascularization: 112.5 versus 139.5 minutes (*p* = 0.158) (Figure 3). Working-hours versus out-of-hours admissions did not differ significantly with regard to favourable functional outcome rate (2/24 versus 15/52; *p* = 0.074, Fisher's exact test) at 90 days.

### 3.1. Development of Procedural Times and Clinical Outcome

Procedural times improved slightly after establishing a 24-hour neuroradiological on-site service in May 2012 (Table 1 and Figure 3). However, both during working-hours admissions and out-of-hours admissions, changes in door-to-image (*p* = 0.535 and *p* = 0.604), image-to-puncture (*p* = 0.209 and *p* = 0.268), puncture-to-revascularization (*p* = 0.538 and *p* = 0.984), and door-to-revascularization times (*p* = 0.205 and *p* = 0.204) failed to reach statistical significance.

In out-of-hours admissions, additional workflow optimization with extensive documentation of procedural times had a significant impact on door-to-image (*p* < 0.001), image-to-puncture (*p* = 0.013), and door-to-revascularization times (*p* = 0.024). Changes in puncture-to-revascularization time failed to reach statistical significance (*p* = 0.259). In working-hours admissions, the same measures resulted in significantly shorter door-to-revascularization times (*p* = 0.026). However, when changes in door-to-image (*p* = 0.061), image-to-puncture (*p* = 0.063), and puncture-to-revascularization times (*p* = 0.096) were regarded separately, these changes failed to reach statistical significance.

In out-of-hours admissions, establishing a fulltime neuroradiological on-site service combined with workflow optimization with extensive documentation of procedural times resulted in significantly improved door-to-image (*p* < 0.001), image-to-puncture (*p* = 0.003), and door-to-revascularization times (*p* = 0.001), whereas puncture-to-revascularization times (*p* = 0.365) did not change significantly. In working-hours admissions all procedural times, including door-to-image (*p* = 0.029), image-to-puncture (*p* = 0.006), puncture-to-revascularization (*p* = 0.045), and door-to-revascularization times (*p* = 0.006), improved significantly during the same period.

## 4. Discussion

With the introduction of a fulltime neuroradiological on-site service, we aimed to accelerate our procedural times and achieved slight improvements. We expected that the out-of-hours presence of a neuroradiologist would result in acceleration of image-to-puncture times and to a lesser degree also door-to-image times. However, a fulltime neuroradiological on-site service resulted only in a nonsignificant improvement of procedural times. A significant acceleration of procedural times could only be achieved when also the interdisciplinary workflow was optimized: in February 2014 we implemented a modified stroke workflow involving all participating parties beginning from the surrounding rescue coordination centres to our stroke unit and intensive care units [16–18]. We modified our chain of information with early notification of the neuroradiologist and the anaesthesiologist in charge of all possible endovascular cases by the neurologist (Figure 1). In order to raise awareness of procedural times, we introduced uniform documentation throughout the complete rescue chain, including extensive documentation of procedural times by both the neurologists and the neuroradiologists (Figure 2). These measures resulted in a highly significant reduction of door-to-image, image-to-puncture, and door-to-revascularization times in out-of-hours admissions. By combining a fulltime neuroradiological on-site service and comparatively simple and inexpensive workflow optimization, we were able to significantly reduce almost all procedural times in both working-hours and out-of-hours admissions without creating a weekend-effect. In particular, we were able to accelerate in-hospital procedures that rely on efficient teamwork (reflected in door-to-image and image-to-puncture times). In working-hours we were also able to accelerate puncture-to-revascularization times, a measure that rather reflects the speed of the actual thrombectomy than organizational workflow. However, we were not able to reduce puncture-to-revascularization times in out-of-hours admissions. This finding is surprising as puncture-to-revascularization times reflect the type of LVO and the interventionalists' experience rather than workflow issues. It is conceivable but unlikely that interventionalists simply take their time in out-of-hours admissions. However, our findings may rather imply that there are organizational issues (i.e., preparation of the procedure and assistance during the procedure) that cannot be overcome with a reduced staff of radiographers and assistants. Identifying specific factors that could improve procedural times should be subject of further studies.

In times of highly effective EST in AIS patients with LVO, it is mandatory to optimize all steps within the stroke rescue chain. Extensive intrahospital workflow optimization with specific standard operating procedures for all disciplines, that is, emergency physicians, neurologists, (neuro)radiologists, and anaesthesiologists, as well as for physicians, nurses, technical assistants, transportation, and registration staff, combined with constant feedback mechanisms, can dramatically improve procedural times and therewith outcome of patients [19]. Likewise, logistic and technical changes, as, for example, one-stop management using the latest generation of flat detector CT, have the potential to improve intrahospital treatment intervals. However, most of these measures are not easy to implement and do not affect the prehospital interval [20]. In this work, we introduced an inexpensive and easy to implement stroke documentation tool that covers the entire stroke rescue chain. It helps to improve treatment intervals and can serve as a quality control tool.

Surprisingly, our measures had no impact on clinical outcome at follow-up. This is unexpected as clinical outcome is time-dependent [21]. The fact that our improved procedural times are not reflected in improved clinical outcome is likely owed by the fact that our inclusion criteria became more ambitious over time, resulting in inclusion of patients with significantly larger infarctions (Table 1), hence less favourable expected benefit. 

*Limitations*. We aimed to investigate the impact of a fulltime neuroradiological on-site service on procedural times in out-of-hours admissions. A major limitation of our study is that our results cannot easily be transferred to any other hospital, given the specific infrastructural and organizational structures in every hospital. A major limitation is also owed to the nature of our study as procedural times are influenced by multiple factors and as a neuroradiological on-site service is no isolated variable that can be analysed independently. We anticipated this issue by including a large number of patients and accounting for various possible covariates.

## 5. Conclusions

Our results imply that a fulltime neuroradiological on-site service accelerates procedural times, but that changes are only significant if there is also workflow optimization of the entire interdisciplinary pre- and intrahospital stroke rescue chain. By combining a fulltime neuroradiological on-site service and workflow optimization via uniform documentation throughout the rescue chain, we were able to significantly reduce almost all procedural times of both working-hours and out-of-hours admissions without creating a weekend effect.

## Figures and Tables

**Table 1:** Overview of included patients. Phase 0: before establishing a 24 h neuroradiological on-site service in May 2012. Phase 1: after introduction of a 24 h neuroradiological on-site service but before workflow optimization with extensive documentation of procedural times in February 2014. Phase 2: after workflow optimization with extensive documentation of procedural times. NIHSS: national institute for health stroke scale; mRS: modified Rankin scale; ASPECTS Alberta stroke program early CT score. ICA: internal carotid artery; MCA: middle cerebral artery; ACA: anterior cerebral artery; VA: vertebral artery; BA: basilar artery; PCA: posterior cerebral artery. IA: intra-arterial. EST: endovascular stroke treatment. Values expressed as means ± standard deviation if not indicated otherwise.

	Phase 0	Phase 1	Phase 2	*p* value
Demographics				
Age (yr) (*n*)	69.8 ± 15.7 (median, 72.9; IQR, 16.5)	71.5 ± 12.7 (median, 72.1 IQR, 18.1)	70.4 ± 14.2 (median, 72.9; IQR, 16.7)	0.673
Male sex (*n*)	52 (54.2%)	63 (50.0%)	43 (43.0%)	0.284

Medical history				
Hypertension (*n*)	69 (71.9%)	100 (79.4%)	83 (82.0%)	0.156
Diabetes (*n*)	26 (27.1%)	22 (17.5%)	35 (35.0%)	0.011^*∗*^
Fat metabolism disorder (*n*)	36 (37.5%)	38 (30.2%)	23 (23.0%)	0.087
Adiposity (*n*)	31 (32.3%)	30 (23.8%)	21 (21.0%)	0.166
Nicotine (*n*)	22 (22.9%)	35 (27.8%)	20 (20.0%)	0.381
Cardiovascular disease (*n*)	49 (51.0%)	69 (54.8%)	29 (29.0%)	<0.001^*∗*^
Atrial fibrillation (*n*)	47 (49.0%)	61 (48.4%)	51 (51.0%)	0.924
Prior stroke (*n*)	16 (16.7%)	23 (18.3%)	19 (19.0%)	0.910
Current antiplatelet use (*n*)	26/93 (28.0%)	34/121 (28.1%)	27/82 (32.9%)	0.711
Current anticoagulant use (*n*)	8 (8.3%)	16 (12.7%)	16 (16.0%)	0.264

Clinical baseline				
NIHSS on admission	17.6 ± 7.0 (median, 17.0; IQR, 8)	17.9 ± 7.2 (median, 18.0; IQR, 6)	17.6 ± 5.5 (median, 19.0; IQR, 3)	0.935
mRS on admission	4.6 ± 0.6 (median, 5.0; IQR, 1)	4.5 ± 0.7 (median, 5.0; IQR, 1)	4.5 ± 0.6 (median, 5.0; IQR, 1)	0.734

Site of vessel occlusion				
Anterior circulation (ICA, MCA, ACA) (*n*)	81 (84.4%)	108 (85.7%)	86 (86.0%)	0.942
Posterior circulation (VA, BA, PCA) (*n*)	15 (15.6%)	18 (14.3%)	14 (14.0%)	
Initial ASPECT score	9.0 ± 1.4 (median, 10.0; IQR, 2)	8.9 ± 1.6 (median, 9.5; IQR, 1)	7.6 ± 3.4 (median, 9.0; IQR, 4)	<0.001^*∗*^

*Time intervals*				
Onset to door (min)	89.7 ± 92.5 (median, 61.0; IQR, 50)	111.3 ± 76.8 (median, 95.0; IQR, 92)	96.9 ± 63.8 (median, 79.5; IQR, 73)	0.247
Door to image (min)	30.6 ± 15.9 (median, 27.0; IQR, 12)	27.81 ± 9.9 (median, 28.0; IQR, 12)	24.0 ± 11.2 (median, 22.0; IQR, 10)	0.001^*∗*^
Image to puncture (min)	66.4 ± 34.0 (median, 60.0; IQR, 33)	56.8 ± 22.3 (median, 53.0; IQR, 55)	47.6 ± 18.6 (median, 46.0; IQR, 23)	<0.001^*∗*^
Puncture to revascularization (min)	87.0 ± 58.9 (median, 64.5; IQR, 68)	77.9 ± 46.2 (median, 67.5; IQR, 62)	71.5 ± 49.9 (median, 54.5; IQR, 55)	0.100
Door to revascularization (min)	188.5 ± 84.4 (median, 171.0; IQR, 86)	162.4 ± 52.6 (median, 156.0; IQR, 72)	150.4 ± 71.8 (median, 129.0; IQR, 66)	0.001^*∗*^
Onset to revascularization (min)	268.0 ± 116.2 (median, 233.5; IQR,121)	265.8 ± 97.8 (median, 254.5; IQR, 106)	239.6 ± 91.1 (median, 216.0; IQR, 118)	0.244

*Interventional procedures*				
Systemic thrombolysis (*n*)	69 (71.9%)	87 (69.0%)	67 (67.0%)	0.759
IA thrombolysis (*n*)	34 (35.4%)	28 (22.2%)	9 (9.0%)	<0.001^*∗*^
Stent retriever (*n*)	77 (80.2%)	118 (93.7%)	97 (97.0%)	<0.001^*∗*^

Outcome				
Successful revascularization (TICI ≥ 2b) (*n*)	80 (83.3%)	104 (82.5%)	86 (86.0%)	0.771
Good clinical outcome (mRS ≤ 2 d90) (*n*)	31/79 (39.2%)	34/114 (29.8%)	18/78 (23.1%)	0.087
Death (d90) (*n*)	24/79 (30.4%)	38/114 (33.3%)	29/78 (37.2%)	0.664

^*∗*^ *p* values < 0.05 were considered significant.
